# Implementing PCR testing in general practice—a qualitative study using normalization process theory

**DOI:** 10.1186/s12913-023-10355-4

**Published:** 2023-11-30

**Authors:** Sinead Shahrzad, Kirubakaran Balasubramaniam, Marius Brostrøm Kousgaard, Trine Thilsing, Jens Søndergaard, Gritt Overbeck

**Affiliations:** 1 Department of Public Health, University of Southern Denmark, Research Unit of General Practice, Odense, Denmark; 2 Department of Public Health, University of Copenhagen, Center for General Practice, Copenhagen, Denmark

**Keywords:** *Implementation*, *POC PCR testing*, *Evaluation*, *Normalization process theory*

## Abstract

**Background:**

The COVID-19 pandemic brought attention to a need for rapid testing of large populations. Experiences from community-based testing settings show that there can be workload difficulties, logistical challenges and socioeconomic downsides to large scale Polymerase Chain Reaction (PCR) testing. Alternative testing arenas have therefore been considered. Rapid point-of-care (POC) PCR test methods have since been developed and could have potential to surveille viral respiratory infections. It is, however, unknown if PCR testing can be successfully implemented routinely in general practice. The aim of this study was to assess factors that enable and inhibit the implementation of point-of-care PCR testing for acute respiratory tract infection in general practice.

**Methods:**

Fourteen general practices in the east Zealand area in Denmark were included in the study and given access to POC PCR testing equipment during a flu season. The participating clinics were initially trained in the use of a POC PCR testing device and then spent 6 weeks testing it. We conducted qualitative interviews with general practitioners (GPs) and their staff, before and after the testing period, specifically focusing on their clinical decision-making and internal collaboration in relation to POC PCR testing. We used normalization process theory to design the interview guides and to analyze the data.

**Results:**

Professionals reported no clinical need for a POC PCR testing device in a non-pandemic clinical setting. Results were delivered faster, but this was only timesaving for the patient and not the GP, who had to perform more tasks.

**Conclusion:**

In its current form, the added diagnostic value of using POC PCR testing in general practice was not sufficient for the professionals to justify the increased work connected to the usage of the diagnostic procedure in daily practice.

**Trial registration:**

n/a.

**Supplementary Information:**

The online version contains supplementary material available at 10.1186/s12913-023-10355-4.

## Background

Since the COVID-19 pandemic in 2020, healthcare systems all around the world have gained a lot of experience with Polymerase Chain Reaction (PCR) testing entire populations. These experiences show, among other things, that testing on such a large scale can be an expensive and troublesome task for healthcare systems [1]. In the case of a future pandemic, it is possible that multiple countries will consider at scale testing again. For this reason, alternative testing set-ups have been considered. In order to lighten the socioeconomic and organizational burden of the healthcare system and patients, implementing PCR testing in general practice could be a potentially successful alternative in future pandemic situations, as well as an overall improvement of diagnostics [2].

In Denmark, 5-min. strep A antigen and C-Reactive Protein (CRP) testing has been the traditional standard point-of-care procedure for examining patients with acute respiratory tract infections (RTI) in primary care. Recently, there has been an increasing interest in adding POC PCR testing for infectious diseases as a standard procedure, since it may not only save time spent for each patient compared to usual PCR testing with specimens sent to a laboratory, but also offer the possibility of diagnosing other infectious agents than current POC tests [2].

Implementing new technology in health care organizations can be challenging, and studying how new technology is embedded in clinical practice and how professionals experience the potential added value is important to policymaking. It is well-known that a positive impact of new health technology relies greatly on the acceptance by its users and whether they are able to integrate it in clinical practice [3, 4]. Increased use of POC testing could potentially be cost effective for health services [5, 6]. However, despite an increasing introduction of POC tests, the experiences with using such technology in general practice has not been studied sufficiently [7]. It is therefore important to understand the different facilitators, barriers and individual and collective efforts involved in implementation.

The aim of this study was to explore how a device for PCR testing (cobas® Liat®) was adopted and experienced by health professionals in general practice through a six-week period, and to identify facilitators and barriers for implementation.

## Method

This was a qualitative interview study performed in general practice.

In reporting this study, we have followed the Consolidated Criteria for REporting Qualitative research (COREQ) checklist [8]. The study is a qualitative study of a quality improvement initiative in independent GP clinics and it included no human experiments or use of human tissue. The University of Southern Denmark (SDU) approved study procedures (reference number: 11.582) and informed consent was obtained from all participants (healthcare professionals) prior to study procedures.

### Setting

**Fig. 1:**
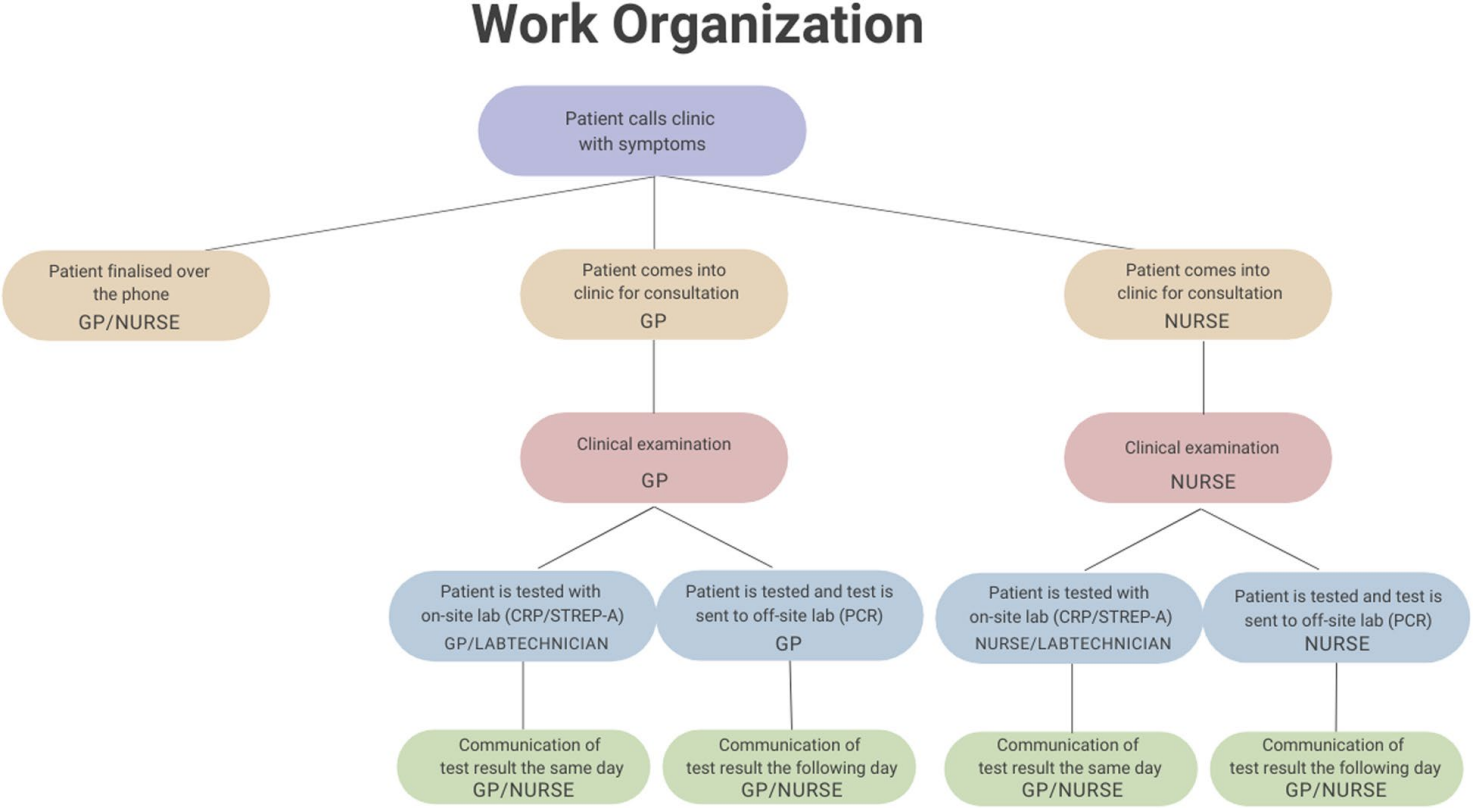
Various work routines involved in the diagnostic process

General practices in Denmark are privately owned companies and are financially incentivized by tax-funded procedural fees. They can be run as collaboration clinics with several independent general practitioners (GPs) and a shared income, as single-handed clinics, or as partnership clinic with shared facilities, staff and separate incomes. The GPs can hire staff, e.g., secretaries, nurses, or laboratory technicians, but are still the ones responsible for treatment. Currently, the diagnosis of a patient with RTI symptoms can be performed in several different ways. Some are resolved over the phone, some in person by a nurse and some take days to finalize due to outsourcing of laboratory tests (Fig. 1). The participating clinics were invited by email and telephone to test the device by Roche Diagnostics (Copenhagen, Denmark), a medical device manufacturer specialized in diagnostics, to resemble a naturalistic clinical device setting.

### Testing device

Cobas® Liat® is a PCR testing device for rapid PCR testing and at the time of this study it had three different test panels for RTIs: SARS-COV-2 & Influenza A/B, Influenza A/B & RSV and Strep A, and therefore allowed the clinics to test for common respiratory tract pathogens in-house. All tests take 15–20 min to complete [9]. This POC PCR testing device has only been used in hospital settings in Denmark until now.

In this study, the Cobas® Liat® device was free to use during the testing period and all expenses related to testing kits and device maintenance were covered by Roche Diagnostics.

### Participant recruitment

To explore implementation in general practice, qualitative, semi-structured interviews were conducted with HCPs(thirteen GPs, one nurse and two lab technicians). Twelve of the participating informants were women, four were men. The clinics were located around the eastern part of Zealand and dealt with both elderly and young patients. There was no relationship or familiarity between researchers and professionals prior to the study. No one else, besides the interviewers and the professionals, were present during the interviews.

Pre-implementation, general information was collected on the participating clinics. Their number of registered patients ranged between 1900 and 7500, depending on whether they were a single-handed practice or a partnership practice, and the number of GPs per practice ranged from 1 to 3.

Each clinic borrowed a device and received an introductory course in its uses and functions by its distributors at Roche Diagnostics. The device was rolled out consecutively, and thereby used at different start and end points, during the trial period from March 2022 to June 2022. These differing start and end points led to some clinics testing the device during a high flu season and others during low flu seasons. Each clinic had the device for six weeks and was otherwise left alone during the trial period. If they ran out of test kits, the professionals requested additional kits free of charge from the distributor. The clinics received 1,000 DKK (equivalent to approximately 140 USD) per hour for the time they spent receiving instruction on how to use the equipment. The instruction took 2 h per clinic. Clinics received 1,000 DKK from University of Southern Denmark for the time spend on interviews.

### Qualitative interviews

Semi-structured qualitative interviews were performed with the professionals in two rounds: pre-implementation and post-implementation. The pre-implementation interviews were necessary to understand the clinics’ usual practice and work division when working with a RTI patient, as well as their use of testing technology and diagnostic tools. The post-implementation interviews were necessary to uncover the relevant changes in practice and experiences with using the devices. All interviews were conducted by SS (MA Medical Anthropologist with special knowledge of health technology) or GO (PhD, implementation scientist) and took place either in the clinics or online, if more convenient for the professionals. Participation in interviews was voluntary.

We developed topic guides for pre- and post-implementation interviews. The guides were based on the four components of normalization process theory (NPT). NPT is a sociological theory that aims to understand how and why new interventions are implemented (or not implemented) in organizational practice by drawing attention to four analytical domains: coherence, cognitive participation, collective action and reflexive monitoring [10, 11] (see supplementary file A). The interviews were audio recorded, or video recorded (in the case of online interviews) and transcribed verbatim by a student assistant. The recordings were not shared with anyone outside the research team. Data saturation was discussed after 10 post-implementation interviews and it was decided to conduct an additional interview to capture possible nuances [12].

The transcribed data was uploaded into Nvivo in Danish for data management and analysis. The data was coded and discussed by researchers SS and GO. The analysis aimed at coding and identifying key implementation efforts, the use of the technology and implementation facilitators and barriers. Throughout the article, anonymized quotes (translated into English) are used illustratively.

## Results

The findings from the interviews are presented based on the four main components of normalization process theory: coherence, cognitive participation, collective action and reflexive monitoring. Out of the fourteen participating clinics, only eleven clinics were available for post-implementation interviews due to time constraints (Table 1). The time-range of pre-implementation interviews was 18:30–51:40 min with a mean duration of 30:32 min. Post-implementation interviews ranged from 11:24–25:52 min with a mean duration of 18 min.

**Table 1:** Overview of participants and test weeks

Clinic no	Type of practice:	Gender	Job title of the HCPs	Years of working experience	Test weeks
1	Partnership practice	Woman Woman	GP and Lab technician	N/A	N/A
2	Partnership practice	Man	GP	7	Week 17–23 2022
3	Partnership practice	Woman	GP	N/A	Week 17–23 2022
4	Collaboration practice	Woman	GP	N/A	Week 14–20 2022
5	Collaboration practice	Man	GP	N/A	Week 14–20 2022
6	Single handed practice	Man	GP	12	Week 13–20 2022
7	Partnership practice	Woman	GP	N/A	Week 14–20 2022
8	Single handed practice	Woman	GP	18	Week 17–23 2022
9	Partnership practice	Woman	GP	7	Week 20–26 2022
10	Single handed practice	Woman Woman	GP and nurse	13	Week 12–18 2022
11	Collaboration practice	Woman	GP	15	Week 13–19 2022
12	Collaboration practice	Woman	GP	N/A	Week 13–19 2022
13	Single handed practice	Man	GP	11	Week 10–16 2022
14	Partnership practice	Woman	Lab technician	N/A	Week 11–17 2022

Below, we sum up the various enablers and critical barriers for implementing the technology for routine use in general practice, which were identified in the study (cf. Table 2).

**Table 2:** Enablers and barriers to implementation

Enablers	Barriers
• Experience with taking and analyzing swab tests • Interest in new diagnostic technology • Introductory course • The ability of the device to identify whether a viral infection was due to a RS virus or COVID-19 • The ability of the device to rule out alternative diagnoses • The patients can receive test results faster	• No serious perceived need for the technology • No important benefits to clinical decision making • Extra work for the HCP • Potential financial costs • The inability of the device to perform more than one test simultaneously in another pandemic situation

### Coherence: making sense of the new technology and its potential

Here we report on the professionals’ understanding of the ‘problem’ at hand, and on their understanding of the device and its potential. We specifically addressed the professionals' understanding of what they were doing differently from their usual practice. Pre-implementation, the participating HCPs reported an overall satisfaction with their current testing procedures, which consisted of Strep-A antigen and CRP testing.

“We are just a clinic that really likes measuring equipment. We like our CRP instruments and all the other instruments we have […]” (GP7, pre-implementation)

While the existing test equipment, consisting of Strep-A antigen test and CRP tests, could respectively rule out the presence of streptococcus bacteria and measure levels of inflammation, the professionals pointed out that these tests cannot offer patients a specific diagnosis which a PCR test can provide by revealing the microorganism likely to cause the condition. For instance, most patients with RTI symptoms do not have a streptococcus infection and the Strep-A test therefore leaves the patient with no specific diagnosis, besides a confirmation of what they do *not* have. Following COVID-19, the HCPs believed that patients had become accustomed to getting a specific name for their condition by the end of the consultation, potentially making the device relevant and useful.

“Yes, because we want to finalize the patient before they head home, you know? So that we won’t have to be on the phone with them the entire following day” (GP8, pre-implementation)

“We are in a time where people want specific answers.” (GP2, pre-implementation)

While HCPs had an interest in rapidly providing a specific diagnosis for the patient's condition, based on what a PCR test can offer in terms of revealing a causal agent for the RTI, such as 'RS-Virus' or 'Influenza A,' they did not previously encounter any serious problems with diagnosing their patients and utilizing the tools currently available. There were no patient complaints about the waiting time for out-of-house laboratory tests and therefore the HCPs did not express any urgent need for permanently adopting the PCR-testing machine.

Some professionals did experience trouble with the device’s buttons being too small while operating it, but in general, the professionals communicated a clear understanding of the device’s functions and usability partly due to the introductory course.

“I think it made sense from the beginning. I think it’s because we were taught in peace and quiet what to do and what it was about.” (NURSE10, post-implementation)

“We thought that it could be nice for the patients to know and get a clarified answer, if they had a type of influenza or if it was Corona.” (GP7, post-implementation)

### Cognitive participation: engagement in the implementation process 

Participants described different reasons for agreeing to try out the new technology. As mentioned above, the HCPs were curious and interested in the technology and in the possibility of being able to give patients a more precise diagnosis. Some GPs also wanted to give their laboratory staff the opportunity to try out alternative diagnosis instruments.

”We have a very eager medical laboratory technologist. She really wants to try out these things as well, and I think it could be exciting to see if it could make a difference for some of the patients, where we think, well, take the flu-season. Here, patients could have the flu, and it could be nice to have a diagnosis relatively fast.” (GP1, pre-implementation)

After becoming familiar with the device, none of the participants reported any disagreement between HCPs or patients in the clinics concerning the device, or that anyone was against the use of it. Staff were generally open to trying it out and learning how to use it, even those not directly participating in patient diagnosis. In general, the participating HCPs were all engaged and involved in the implementation process. Creating a new routine for the use of the device was a crucial element in getting the clinic staff to operate the PCR device.

“I would say that we all used it equally. We reminded each other that it would be a good idea to use it, we talked about it, supervised or gave feedback to each other. And then I think we made sure everyone was in on it and that they could have access to it.” (NURSE10, post-implementation)

“The staff had to be reminded about the device during the first two weeks, but subsequently it became a part of their routine armamentarium. It took some time to integrate it in the routines, especially since it is a somewhat lengthy test, compared to what we're accustomed to.” (GP2, post-implementation)

### Collective action: changes in procedures and routines

Given the late introduction of the device to some clinics, not all clinics were using the device during the season where most patients with the flu or flu-like symptoms were seen. Some HCPs were therefore less likely to have used the device, as only few patients with symptoms of RTI would show up. Some clinics tested 7–10 patients a day, while others tested 30 patients in total in the span of 6 weeks.

After testing the device for 6 weeks, most professionals reported that the device was easy to operate, and instructions were easy to follow due to the training they had received. They also noted that the device was especially relevant for rapid making a COVID-19 testing which could then be dealt with swiftly.

The HCPs only reported on minor changes in their consultations compared to how they had described their diagnostic decision-making process prior to adopting the device. In general, their prior decision-making process consisted of receiving patients with RTI symptoms, recording patient history and doing an objective assessment, deciding what diagnostic tool to use, take the relevant tests and sending the tests to a laboratory, when necessary, and finally completing the consultation. The diagnostic process had not changed drastically post-implementation. The HCPs reported having the same clinical procedures (history recording, objective assessment and possibly CRP and/or Strep-A) and outcomes compared to pre-implementation, with shorter time to diagnosis, being the only noticeable factor for the patient. For the HCPs, the procedure gave extra work, saved no time and reportedly did not lead to any different treatment. However, in the case of wanting to use a Strep-A test for a possible bacterial infection, time was not being saved using the PCR testing machine, since their usual Strep-A test only takes 5-min.

“So, in that way, we do receive the results, about whether it could be influenza, faster. However, the Strep-A is faster without the [PCR] machine. It is only 5-minutes, right? So, it can’t really replace that.” (GP8, post-implementation)

A new routine, concerning the procedure for contacting patients after they had been tested, also had to be incorporated into the daily schedule of the clinics.

“We just sent the patients home and then I sent them a text message afterwards because of logistical reasons. The device was connected to a computer, which is in our lunchroom and our clinic is above it. So, to me, it was easier to collect the tests during the morning consultations, and I then run the tests while having lunch.” (GP7, post-implementation)

### The new routines were, however, adapted relatively quickly

The HCPs reported that their prior knowledge of RTI symptoms influenced how often they used the device. If the observed symptoms were likely to be due to a bacterial infection, a common 5-min Strep-A antigen test would be preferred over the 20-min-long PCR test. If the Strep-A antigen test was negative, the device would in some cases be used to either double-check or check for a viral infection.

“We can’t use (20 min. PCR Strep-A test) for much, but I’ve used it once. If I got a negative Strep-A, as a quick test, but it clinically looked a lot like a bacterial throat infection; I would then use the liat [the device] to double test. That is basically the only time I used it.” (GP12, post-implementation)

If a quick 5 min. CRP test also showed a low value, the PCR test could be used to check for other reasons for the patient’s discomfort.

“No, I think that we just tested those who had unspecified respiratory tract symptoms, where they may not have scored as high on the CRP, we thought it might be nice for them to find out if it was a flu or if there was something else, like corona.” (GP7, post-implementation)

The PCR test could also be used to rule different diagnoses out, e.g., if the GP wanted to make sure that the patient was not suffering from bacterial infections such as tonsillitis.

“I think that we essentially used it on patients with long-lasting symptoms, like 5-10 days and no improvement, or with people calling with respiratory symptoms, or with a relevant history of symptoms that made us think a Strep-A would be insufficient. Or young people, where one would think; is it a Strep-A, or do they have mononucleosis? Then you could rule things out this way. So, no specific age groups, not more men than women or anything like that.” (GP13, post-implementation)

“It was primarily used on the patients we received during our acute appointments. Those who had a sore throat, fever, where it was a bit unsure, what was wrong with them.” (GP11, post-implementation)

While most clinics used the test on patients of all ages and genders, a single clinic reported not using the PCR test on small children, since they deemed it too invasive.

“For me, it was mostly used on adults. I had a few young children, but I did not dare to start swabbing them in their nose. I chose to stick with adults and there were a lot of them anyways. The ‘flu momentum’ was starting to slow down, so I chose to go for people with relevant symptoms that showed possible viral infections.” (GP4, post-implementation)

### Reflexive monitoring: assessments of the new test routine

In regard to beneficial changes in their clinical decision-making, the HCPs reported that the most important contribution of implementing the device was the ability to uncover whether a viral infection was due to a RS virus or COVID-19, and that this could be ‘nice’ for the patients to know. Apart from this, the device did not affect their diagnostic process, which primarily involved a clinical examination of the patient. According to the HCPs, the POC PCR testing machine did not outperform clinical experience and pre-existing test options.

A big drawback of the device was the inability to perform more than one test simultaneously. If another pandemic were to occur, the device would not have the capacity to test the increased number of patients every day. The HCPs also reported that their clinics did not have the physical capacity to receive multiple possible COVID-19 infected patients simultaneously. In-house COVID-19 surveillance would therefore not be possible in practice, according to the HCPs’ experiences with both COVID-19 and the PCR testing machine.

“The pros are that we get an answer faster than if we were to send the tests away. But with a bacterial infection, you cannot use this device in general practice. It will take too long, the patient will have to wait, and we just need them to get out as soon as possible. And with our regular quick Strep-A test, it already works. So, the cobas Liat Strep-A is a no go. But the other two [SARS-COV-2 & Influenza A/B, Influenza A/B & RSV] are fine, they are a ‘nice to have’ [i.e., not a real need to have]”. (GP8, post-implementation)

So according to the HCPs, the test results from the new device had little impact on their clinical decisions and added only little diagnostic value and no alternative treatment options. Some HCPs reported only using the device as frequently as they had because of their commitment to the project. They thought that they would use the device less frequently if it was to become a part of usual practice. A few HCPs also brought attention to the financial costs of using the device routinely and expressed that coverage of these costs would be important if they were to consider adopting the device in usual practice.

## Discussion

This study was conducted after COVID-19 had subsided in Denmark, and the objective was to investigate experiences of trying out a new PCR testing device in general practice and to identify enablers and barriers for implementation in a non-pandemic situation. Fourteen general practice clinics adopted a 15–20 min. POC PCR testing machine for upper respiratory tract infections and used the device for 6 weeks.

The device was initially easy to integrate due to the following enabling factors. The participating HCPs were curious and interested in testing with a new POC PCR testing machine. A positive disposition was initially enabling for the integration of the device [13]. Furthermore, the manual setup of the device was easy to understand, and the HCPs were quick to learn its functions having received an introductory course. Also, the HCPs had previous experience with swabbing and analyzing swab tests from e.g., Strep-A antigen testing and hence the technology was compatible with pre-existing knowledge and competencies in the practice. These pre-conditions and activities promoted coherence in terms of understanding the purpose and functions of the new technology; an otherwise common obstacle to implementation of health innovations [11, 14–16]. However, despite this initial positive reception, some important barriers were identified. Besides their professional interest and curiosity, there was a low perception of any real need for an in-house POC PCR testing machine. Prior to the implementation, the HCPs did not express any problems with their current diagnostic procedures or any requests for change, which is often an important prerequisite for implementing new procedures [17, 18]. Furthermore, the procedures associated with the new device (collecting swab tests, placing them in the machine, waiting for the result and then contacting the patient with the result) also proved to create more work for the HCPs. So although using the device could provide a faster test result for the patient than previously, the HCPs had to spend more time on testing than before, which hampered willingness to implement the device in routine practice [19].

Overall, the results suggest that the observed barriers are more influential than the enablers, when considering the potential for adopting and implementing the technology more widely in general practice in a non-pandemic situation [20, 21].

The 15–20-min waiting time associated with the device is an extra step in an otherwise tried and normalized testing procedure in general practice, where the PCR tests are usually sent to an out-of-house laboratory. While the laboratory results are received at a later time, the HCPs have to do less work and less frequent testing, which is important for the clinical workload [20, 22]. The financial costs may also inhibit the implementation of POC testing devices in routine practice, and future research should include a health economic analysis of adopting the testing devise in general practice [23, 24].

Our study also thoroughly explored the practical challenges that clinicians expected to be associated with the use of the new device in an isolated pandemic situation. If another pandemic were to occur, this device would not be suitable due to logistical challenges. Overall, Scandinavian health care systems experienced less problems than many other health care systems during the Covid pandemic; there was sufficient personal protective equipment (PPE) for health professionals and an efficient, but expensive, testing system was established outside general practice [25–27]. Other healthcare systems saw a range of problems that could be addressed by more availability of POC-testing [28, 29].

### Strengths and limitations

This study used a qualitative approach, which allowed us to understand the enablers and barriers related to integrating a new diagnostic technology in clinical practice. The study was carried out in fourteen clinics that had volunteered to test the device. The findings might therefore reflect a more positive attitude towards the device than what might be the case among the larger population of HCPs in general practice. In addition, the clinics in this study did not have to bear the financial costs of acquiring and using the device which would be an important issue in considerations about adopting the device in routine practice.

Since the GPs are the ones making decisions about adopting new technology in their clinics and since some staff are also involved in using the technology, this study focused solely on the experiences of the professionals. However, this focus fails to acknowledge the experiences of the patients. Evidence regarding positive patient experiences and patient behavior in relation to the technology could potentially be an enabler for adoption [30].

## Conclusion

This study set off to examine the potential of using a new technology for diagnosing respiratory tract infections in Danish general practice. A POC PCR test method was introduced in 14 clinics for a six-week testing period. Despite initial interest in the new test device, the results suggest that the technology, in its current form, does not show promise of widespread adoption and implementation in general practice in a non-pandemic situation. Reducing the time patients spend waiting for a diagnostic answer was not sufficient to convince the clinicians to implement the technology on a permanent basis due to the additional tasks required when using the technology and the fact that using the device did not alter the clinical decision making. Additionally, it was not perceived by the professionals to add sufficient clinical value to everyday practice. Future attempts to introduce new testing methods should consider the balance between clinical workload and clinical value.

### Supplementary Information

**Additional file 1:** Consolidated criteria for reporting qualitative studies (COREQ): 32-item checklist. **Supplementary file A.** Interview topic guides for pre and post interviews

## Data Availability

Data may be made available through formal data sharing agreement with the authors’ institution on reasonable request.

## Abbreviations

HCP: Healthcare professional
GP: General practitioner
POC: Point-of-care
PCR: Polymerase chain reaction
