# Supplementary material for: Implementing PCR testing in general practice—a qualitative study using normalization process theory
Source: BMC Health Serv Res. 2023 Nov 30;23:1325. doi: 10.1186/s12913-023-10355-4 (PMC10687998; doi:10.1186/s12913-023-10355-4)
Supplement: Supplementary file 1 — Additional file 1: Consolidated criteria for reporting qualitative studies (COREQ): 32-item checklist. Supplementary file A. Interview topic guides for pre and post interviews [file 12913_2023_10355_MOESM1_ESM.docx]

**Supplementary file A: Interview topic guides for pre and post interviews**

Interview Guide: PRE-intervention

The interview will cover

- Descriptions of the clinic's current procedure in relation to how they diagnose patients with respiratory symptoms.
- Relevance/perspectives on POC in practice

General information

Clinic type (solo practice?, partnership practice?, community practice?)

Number of patients

Employees and organization of work

Seniority

Symptoms and diagnosis approach

Try to describe a typical patient with respiratory tract infection (RTI) (adult + child)

Try to describe the typical procedure for a patient with respiratory symptoms.

What do you usually do to find out if the PT has an acute respiratory tract infection (RTI)?

Do you think it is easy to make the correct diagnosis?

Is it easy to delineate acute RTI from other conditions?

What diagnostic tools do you use? [NPT: Coll Act]

How do you think it works? [Coll Act]

What types of treatment do you offer patients with RTI?

What influences your treatment decision?

Describe your current workflow from a patient with RTI symptoms comes in the door.

Try to describe an easy course with an RTI patient and a difficult course.

What role does a practice nurse play in the diagnosis?

What role does the clinical microbiology department (at the hospital) play in the diagnosis?

How would you assess the results/usefulness of the treatment that is currently given to patients with a RTI?

What role does play in the diagnosis?

How would you assess the results/usefulness of the treatment that is currently given to patients with a RTI

Reasons to try the PCR rapid testing machine / (NPT cognitive participation)

Why did you decide to participate in testing out the device?

How do you expect it to influence the current workflow in the clinic?

How will it affect your clinical decision-making?

Who will be using the testing device?

The testing device could contribute to the monitoring of future virus epidemics – what do you think about contributing to that?

Is there anything, that you would like to add?

Interview Guide : Post-intervention
The interview will cover the clinic's experiences with using the rapid testing machine

[Coherence]

When did you start using the rapid testing machine?

How were you instructed in its use?

What was easy and what was difficult about the initial instruction from the company (Roche Diagnostics)?

[Cognitive participation]

Was it easy to get the staff in the clinic to use the device?

Did anyone use it more than others did?

[Collective action]

Which patients did you end up testing with the rapid PCR test? (Children, adults, the worried, etc.)

Did the patients stay and wait for an answer or did you call them afterwards?

Try to describe the handling of the machine itself: was there anything difficult or time-consuming?

When you had done a test, did you go out and read the result or did you have it sent directly to your computer? (via a software from the company (Roche Diagnostics))

Who was responsible for contacting the company when you ran out of test kits?

Did you have any problems contacting the company?

[Reflexive monitoring]

Did the rapid PCR testing give your clinical assessment something extra – something you could not already get with the existing machines in the clinic?

Can you see it being used as a tool for epidemic surveillance in a possible future epidemic situation?

Overall, what are the advantages and disadvantages of the rapid PCR testing?
